# The Peridental Structures of Equine Cheek Teeth: An Age-Dependent Anatomical Study of Maxilla and Mandible

**DOI:** 10.3390/ani16142225

**Published:** 2026-07-18

**Authors:** Sarah Fewson, Matthias Lüpke, Maren Hellige, Hermann Seifert, Astrid Bienert-Zeit, Carsten Staszyk

**Affiliations:** 1 Institute for General Radiology and Medical Physics, University of Veterinary Medicine Hannover, Foundation, 30173 Hannover, Germany; matthias.luepke@tiho-hannover.de (M.L.); hermann.seifert@tiho-hannover.de (H.S.); 2 Clinic for Horses, University of Veterinary Medicine Hannover, Foundation, 30559 Hannover, Germany; maren.hellige@tiho-hannover.de; 3 Equine Clinic Burg Müggenhausen GmbH, 53919 Weilerswist-Müggenhausen, Germany; bienert-zeit@pferde-klinik.de; 4 Institute of Veterinary-Anatomy, -Histology and -Embryology, Faculty of Veterinary Medicine, Justus-Liebig-University Giessen, 35392 Giessen, Germany; carsten.staszyk@vetmed.uni-giessen.de

**Keywords:** equine dentistry, cheek teeth, anatomy, morphometry, peridental region, age-related changes, dental disease, sequester, horse, alveolar bone proper

## Abstract

Sequestration is the most common complication following equine cheek tooth extraction, occurring predominantly in the mandible of younger horses. Extraction-related trauma to the peridental region may impair healing. Despite its clinical relevance, detailed knowledge of peridental anatomy remains limited. This study investigated age-related differences in the peridental region of maxillary and mandibular equine cheek teeth 07 to 09. The peridental region of 24 cheek teeth rows from six cadaver heads was sectioned horizontally at approximately 10 mm intervals. Sections were photographed and the proportion of adjacent structures (compact bone, spongy bone, maxillary sinus, nasal cavity) was quantified within a 1 mm zone of the peridental gap. Horses were assigned to three age groups. Maxillary teeth were surrounded by approximately 50% compact bone, while mandibular teeth showed a higher proportion of compact bone occlusally (60.3%) that decreased apically (to 23.7%). The proportion of compact bone generally increased with age. This descriptive anatomical study provides a novel anatomical characterization of the equine peridental region. The higher proportion of compact bone in the mandible suggests reduced bleeding into the empty alveolus. This could impair wound healing and reduce resorption capacity for bone fragments. This may explain the increased risk of sequestration.

## Simple Summary

Cheek tooth removal in horses may lead to severe complications. Sequestration of the alveolar bone, where a fragment of dead bone separates from surrounding healthy bone, is observed particularly following the extraction of younger, and therefore longer mandibular cheek teeth. The bony structures (i.e., alveolar bone proper, spongy bone, compact bone) surrounding a tooth are exposed to high mechanical forces during tooth extraction and are inevitably damaged. Subsequent insufficient bleeding into the empty alveolus impairs the healing process and leads to complications, including sequestration of the remaining alveolar bone. Spongy bone provides sufficient bleeding after extraction. We hypothesize that the higher proportion of compact bone results in reduced bleeding into the alveolus and that compact bone fragments are resorbed less efficiently. These factors may increase the risk of sequestration. In this study, we examined whether differences in the arrangement of the peridental bony structures can explain why the alveoli of younger mandibular cheek teeth are predisposed to develop sequestration following tooth extraction. We discovered that mandibular cheek teeth were surrounded by more compact bone compared to maxillary cheek teeth. The proportion of compact bone generally increased with age. Age-related differences in compact bone distribution cannot solely explain the higher occurrence of sequestration.

## 1. Introduction

Complications following the extraction of equine cheek teeth are clinically relevant in equine dentistry. Therefore, a detailed understanding of the anatomical and structural characteristics of the alveolar bone proper (ABP) and the surrounding tissue is essential.

The ABP is a thin (0.1–0.4 mm), perforated bone plate that lines the dental alveolus [1,2,3,4]. It is referred to as “bundle bone” when describing its histological characteristics, as “lamina cribriformis” based on its macroscopically perforated, sieve-like morphology, and as “lamina dura” in diagnostic imaging [1,5]. The structure is located in the alveolar process of the maxilla and mandible, which together define the tooth-bearing regions of the jaw [1,2]. The ABP and alveolar process are summarized as alveolar bone [4]. The ABP constitutes a component of the periodontium, anchoring the periodontal ligament (PDL) fibers [1,2,4]. Therefore, it is necessary for the stable fixation of the teeth and helps to absorb masticatory forces acting on the teeth [1,2,4].

The ABP is surrounded by spongy bone that is rich in fat and blood vessels. These numerous blood vessels pass through the highly perforated ABP and enter the periodontal space to supply nutrients to the cells of the periodontal ligament and cementum. Peripherally, the spongy bone is adjacent to compact bone, which forms the outer boundaries of the tooth-bearing regions of the jaw. In the maxilla, the compact bone may also form the bony boundary toward the maxillary sinus or nasal cavity. This basic structural principle exhibits local variations, particularly in the thickness of the spongy and compact bone. In certain regions, the ABP is pressed against and fuses with the peripheral compact bone, resulting in the absence of intervening spongy bone [1,2].

Especially in horses, the anatomical variability is further complicated by age-related changes of the tooth. In response to the progressive shortening of the tooth, combined with a simultaneous, steady eruptive movement, the peridental environment changes continuously. During this process, the ABP constantly adapts to the shape of the tooth, physiological tooth movements [1,2] and mechanical forces [2,6], ensuring that the ABP always forms a tight fit around the tooth. Furthermore, continuous tooth eruption and apical apposition of the tooth require ongoing structural adaptations of the ABP, a phenomenon not observed in humans to this extent [2,7].

In addition to the physiological remodeling processes, the peridental region is exposed to intense mechanical forces during the manual extraction of cheek teeth. This not only results in the disruption of the periodontal collagen fiber network, but also in the partial fragmentation and avulsion of the ABP. During the healing process, which can last several months, the emptied alveolus is subsequently filled with a spongy bone material and undergoes re-epithelialization toward the oral cavity [8,9,10,11,12,13,14,15]. Sufficient bleeding into the alveolus following extraction is an essential requirement for adequate wound healing [8,9,10,11,12,13,14,15]. Alveolar healing is dependent on a variety of factors, including the resorption of soft tissue and bone debris resulting from the extraction process, as well as adequate immunological control of infectious agents within the alveolus [9,13,14,15].

Dental extraction is a routinely performed procedure in horses with diseased teeth. Cheek teeth extraction is a challenging procedure with significant risk of post-extraction complications depending on the extraction method [16,17]. Post-extraction complications occur more frequently in younger horses, with reported mean ages ranging from 7.3 to 9.9 years [18,19]. These are most commonly observed following the extraction of mandibular cheek teeth, particularly at tooth positions 07 and 09 [18] or 06 to 08 [19]. Sequestration has been reported as the most prevalent complication following dental extraction [18,19]. A sequestrum is defined as a demarcated fragment of necrotic bone that is separated from the surrounding vital bone resulting from the pathological process of sequestration [20,21,22]. Based on these observations, it was hypothesized that the arrangement of the peridental tissues may influence alveolar healing and/or promote the formation of bone sequestra. Regions in which compact bone is adjacent to the ABP exhibit reduced bleeding into the alveolus following tooth extraction compared to more vascularized bony structures, including spongy bone, the mucosa of the maxillary sinus and that of the nasal cavity. These variations may explain the higher incidence of post-extraction complications in the mandible of younger horses.

The objective of this study was to investigate potential anatomical variations in the structural organization of the peridental region between equine maxillary and mandibular cheek tooth rows. For this purpose, the relative distribution of compact bone and more vascularized bony structures was quantified. We hypothesized that the structure of the peridental region differs between the maxilla and mandible and is subjected to age-related changes.

## 2. Materials and Methods

Samples were collected from six horses aged 5 to 18 years. The horses were either euthanized at the Clinic for Horses (University of Veterinary Medicine Hannover, Foundation, Hannover, Germany) for reasons unrelated to dental disease or obtained from a slaughterhouse following captive bolt stunning and exsanguination. All procedures were conducted in accordance with the approval of the institutional ethics committee of the University of Veterinary Medicine Hannover, Foundation, Germany. The Breed (predominantly warmbloods), age, and gender were recorded. Age of horses were determined based on horse passports and dental age estimation [7]. The heads were disarticulated and cooled. Within 48 h, a dental examination, X-rays of the maxillary and mandibular cheek teeth (parameters: 73 kV, 7.6 mAs, imaging view: hemisphere model maxilla: 90°/+30°, mandible: 90°/−45°, Gierth HF 1000, Gierth X-ray International GmbH, Riesa, Germany), and a computed tomography (CT) were performed (parameters: 140 kV, 1.5 mm slice thickness, matrix: 1024 × 1024, imaging view: reconstructed sagittal section, BrillianceTM CT Big Bore Oncology, Philips Medical Systems International B.V., Best, The Netherlands). The heads and the radiological images were independently evaluated by two observers (SF and ABZ) to exclude heads with dental pathologies (e.g., alveolitis, tooth-associated sinusitis, tooth or bone fractures).

The heads were categorized into three different age groups according to the age-related changes of equine cheek tooth development [2,23]. The grouping was designed to reflect major processes of tooth maturation and ageing associated with continuous eruption, wear, root development, and age-related modifications of the apical region [2,23].

- Group 1 (two heads): 5–9 years
- Group 2 (two heads): 10–15 years
- Group 3 (two heads): >15 years

### 2.1. Preparation of Anatomical Sections

**Figure 1:**
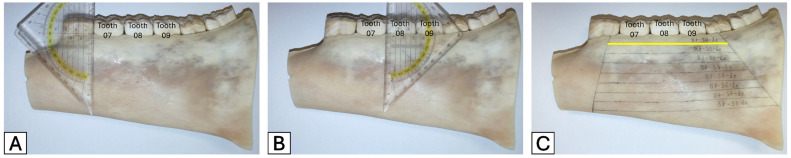
Location of the cutting lines on the buccal side of the mandible, which are used to prepare horizontal sections of the mandibular cheek teeth 07–09. (**A**) Position of the set square on the mesial edge of tooth 07 to identify a marker point at a distance of 10 mm apical of the alveolar rim. The zero point of the set square was placed at the most occlusal point of the alveolar ridge. (**B**) Position of the set square on the distal edge of tooth 07 to identify a marker point at a distance of 10 mm apical of the alveolar rim. The zero point of the set square was placed at the most occlusal point of the alveolar ridge. (**C**) The yellow line results from connecting all marker points at a distance of 10 mm apical to the alveolar rim of cheek teeth 07 to 09. Further parallel lines were drawn at equidistant intervals of 10 mm in the apical direction. The oblique vertical lines indicate the boundaries of the section containing cheek teeth 07 to 09 to reduce the length of horizontal cuts.

**Figure 2:**
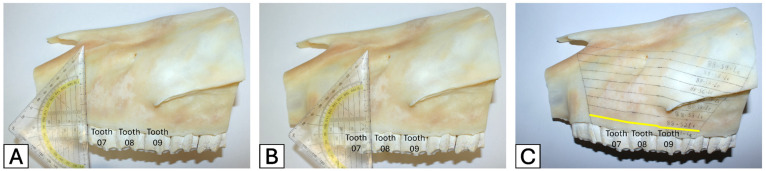
Location of the cutting lines on the buccal side of the maxilla, which are used to prepare horizontal sections of the maxillary cheek teeth 07–09. (**A**) Position of the set square on the mesial edge of tooth 07 to identify a marker point at a distance of 10 mm apical of the alveolar rim. The zero point of the set square was placed at the most occlusal point of the alveolar ridge. (**B**) Position of the set square on the distal edge of tooth 07 to identify a marker point at a distance of 10 mm apical of the alveolar rim. The zero point of the set square was placed at the most occlusal point of the alveolar ridge. (**C**) The yellow line results from connecting all marker points at a distance of 10 mm apical to the alveolar rim of cheek teeth 07 to 09. Further parallel lines were drawn at equidistant intervals of 10 mm in the apical direction. The oblique vertical lines indicate the boundaries of the section containing cheek teeth 07 to 09 to reduce the length of horizontal cuts.

Following the examination, the heads were frozen at −20 °C. The maxillary and mandibular cheek teeth rows were dissected from the heads using an electric band saw (L420, Kolbe GmbH, Elchingen, Germany). The collected specimens were then macerated and bleached in 3% hydrogen peroxide solution for six hours. Subsequently, cutting lines were marked on the buccal surface of the maxilla and mandible to guide sectioning of approximately 10 mm thick horizontal sections from the alveolar ridge to the apical region. The corresponding cutting lines are shown in Figure 1 and Figure 2. The study included maxillary and mandibular cheek teeth 07 to 09 from both the right and left sides. The teeth were numbered according to the modified triadan system [24]. For data analysis, tooth numbers were simplified to 07, 08, and 09, and no differentiation between left and right sides was made. Differentiation between maxillary and mandibular cheek teeth was used to indicate the anatomical location.

**Figure 3:**
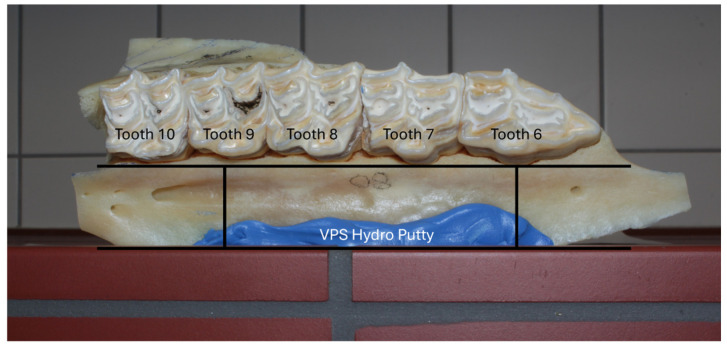
Positioning of the maxilla to ensure horizontally oriented sections (occlusal view).

To ensure straight horizontal cuts, the maxilla was stabilized palatally using VPS Hydro Putty (Henry Schein Inc., Melville, NY, USA) so that the occlusopalatal edge of the cheek teeth 07 to 09 was parallel to the table surface (Figure 3).

Each prepared maxilla and mandible were placed in a water-filled plastic bag with the oral side facing down, sealed, and frozen at −18 °C for 24 h. After freezing, the jaws embedded in ice blocks were sectioned along the marked lines using a diamond-coated, water-cooled, micro band saw (MBS 240/E, Proxxon SA, Wecker, Luxembourg).

The obtained horizontal sections were photographed using a digital camera (Canon EOS 700D, Canon Inc., Tokyo, Japan) mounted on a tripod under consistent lighting. Graph paper was included in each image to provide a scale for subsequent length measurements.

**Figure 4:**
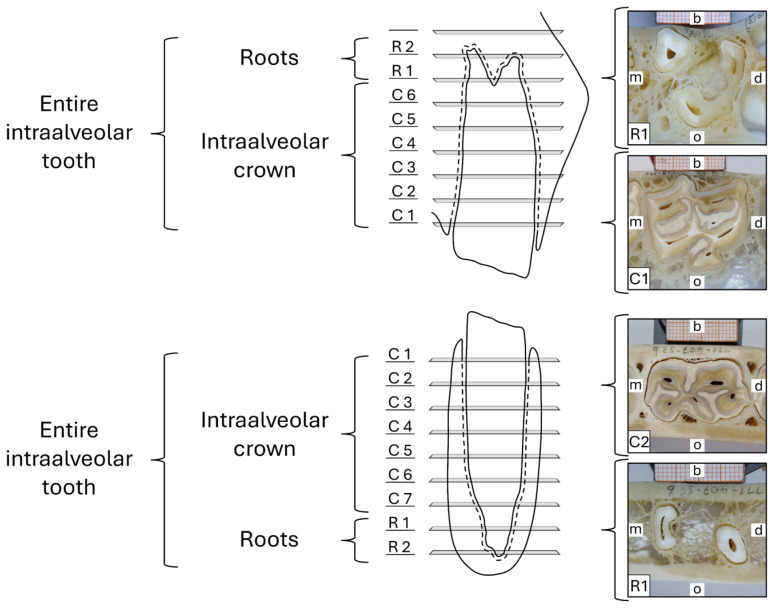
Schematic illustration of the equine maxillary and mandibular cheek teeth shows subdivision into the intraalveolar crown (C1–C7) and the roots (R1, R2). Exemplary macroscopic horizontal sections illustrate the appearance of the obtained photographs of individual section preparations. The intraalveolar crown including the reserve crown and bi- or trifurcation. The roots refer to the apical area of the tooth, where the reserve crown splits into mesial and distal roots in mandibular teeth, or into mesiobuccal (mesial), distobuccal (distal), and palatal roots in maxillary teeth. The entire intraalveolar tooth is defined as the combined sum of the intraalveolar crown and roots. The palatal root is not visible in the illustrated sections and is therefore not labelled. Abbreviations: b = buccal, o = oral, m = mesial, d = distal.

**Figure 5:**
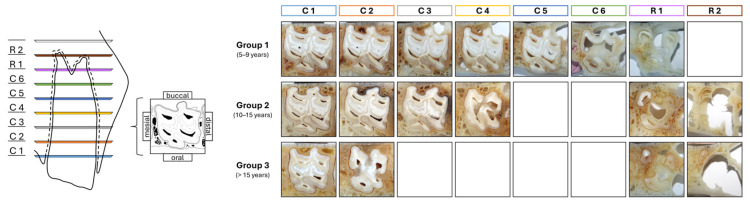
Representative horizontal section of the left equine maxillary cheek tooth 08 across the three age groups. Tooth sections are displayed from left (occlusal) to right (apical): C1–C6 = intraalveolar crown, R1, R2 = roots. The schematic tooth (**left**) illustrates the location of horizontal section. The schematic horizontal tooth section (**middle**) indicates anatomical orientation (buccal, oral, distal, mesial) corresponding to the horizontal sections shown on the (**right**) anatomical horizontal tooth sections. The apparent empty fields are indicative of disparities in age-related shortening of tooth length. In shorter teeth, fewer crown sections are present, and root structures become visible earlier, resulting in an apparent discontinuation of corresponding section levels across rows. Consequently, not all section levels are present in each tooth.

**Figure 6:**
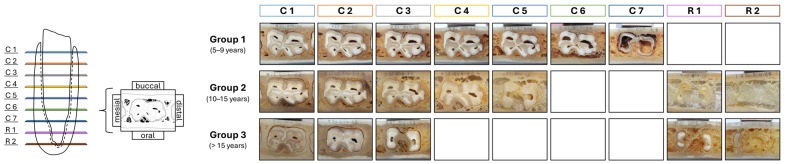
Representative horizontal section of the right equine mandibular cheek tooth 08 across the three age groups. Tooth sections are displayed from left (occlusal) to right (apical): C1–C7 = intraalveolar crown, R1, R2 = roots. The schematic tooth (**left**) illustrates the location of horizontal section. The schematic horizontal tooth section (**middle**) indicates anatomical orientation (buccal, oral, distal, mesial) corresponding to the horizontal sections shown on the (**right**) anatomical horizontal tooth sections. The apparent empty fields are indicative of disparities in age-related shortening of tooth length. In shorter teeth, fewer crown sections are present, and root structures become visible earlier, resulting in an apparent discontinuation of corresponding section levels across rows. Consequently, not all section levels are present in each tooth.

For each tooth, the anatomical structure adjacent to the ABP was assessed and recorded. The tooth was differentiated into the area surrounding the intraalveolar crown and the roots (Figure 4, Figure 5 and Figure 6). In individual maxillary cheek teeth sections, the separation into the mesiobuccal, distobuccal, and palatal root was incomplete, resulting in a mesiobuccal and a fused buccodistopalatal root. These regions were assigned to the root segment.

### 2.2. Analysis of Peridental Structures

**Figure 7:**
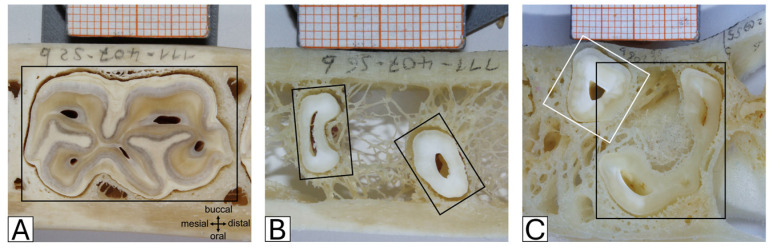
Geometric construction of rectangles around the alveolar bone proper in different regions of prepared horizontal cheek tooth sections. (**A**) rectangle (black) construction around intraalveolar crown of right mandibular cheek tooth 07; (**B**) rectangle (black) constructions around the mesial and distal root of right mandibular cheek tooth 07; (**C**) rectangle construction at the trifurcation level around the mesiobuccal root (white) and the buccodistopalatal root (black) of left maxillary cheek tooth 08. The depicted teeth were from Group 2.

Digital photographs of the sections were analyzed using the software ImageJ (version: ImageJ 1.54p, Wayne Rasband, National Institute of Mental Health, Bethesda, MD, USA). A reference scale was determined by the “Set Scale” tool. Each section image was prepared as follows: a rectangular box was constructed around each intraalveolar crown such that the edges of this rectangle touched the tooth-near surfaces of the ABP (buccal, oral, mesial, distal) at a single point without intersecting them (Figure 7A). The rectangle was constructed according to the same principle at the roots but aligned along the main axis of the root orientation (Figure 7B). If a root was apically divided into two root segments within a section, each segment was measured separately, and the resulting measurements were added for analysis.

**Figure 8:**
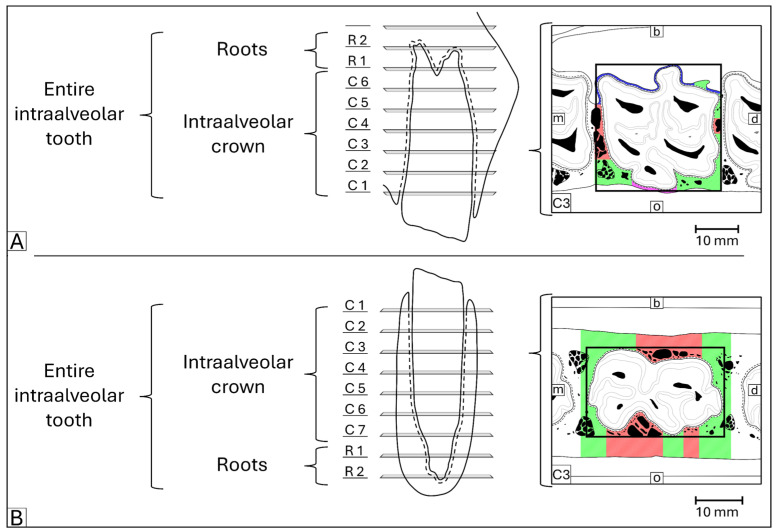
Schematic representation of the anatomical horizontal sections and analysis of the peridental adjacent bone structures. The cheek teeth were subdivided into the intraalveolar crown (sections C1–C7) and the roots (sections R1, R2). Section C3 from both the maxillary (**A**) and mandibular (**B**) tooth is shown on the right. The black-framed rectangle illustrates the geometric simplification of the anatomical structure of the alveolar bone proper. The color-coded areas represent the different peridental adjacent structures: green = compact bone, red = spongy bone, blue = maxillary sinus, pink = nasal cavity. Abbreviations: b = buccal, o = oral, m = mesial, d = distal.

Starting from the tooth-near edge of the ABP, the adjacent structures were examined at a distance of 1 mm toward the tooth-far side. For this purpose, a circular region of interest (ROI) with a diameter of 1 mm was constructed in ImageJ (version: ImageJ 1.54p). The following four structures were distinguished (Figure 8):

- Spongy bone: defined as a region containing three or more pores that can vary in size, but are located less than 1 mm apart, or a single pore with a diameter of ≥2 mm.
- Compact bone: solid bony plate, which does not meet the criteria for spongy bone with a thickness of ≥1 mm. Thinner regions are allowed at the margins of the bone.
- Maxillary sinus: air-filled cavities within the skull bones.
- Nasal cavity: air-filled space inside the nose.

To reliably differentiate between spongy bone, maxillary sinus, and nasal cavity in ambiguous maxillary sections, the sections were additionally evaluated using corresponding CT images.

**Figure 9:**
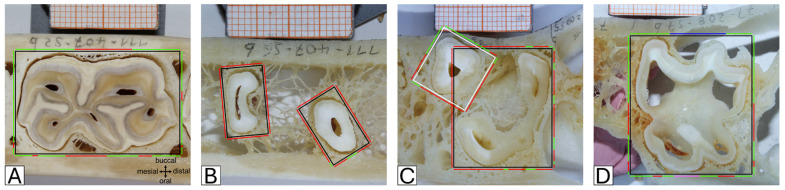
Representative depiction of structures adjacent to the alveolar bone proper in different horizontal tooth sections. (**A**) right mandibular tooth 07, section C1, Group 2; (**B**) right mandibular tooth 07, section R2, Group 2; (**C**) left maxillary tooth 08, section R1, Group 2; (**D**) left maxillary tooth 08, section C6, Group 1. Brown/white rectangles = simplification of anatomical dimensions of alveolus; green lines = compact bone; red lines = spongy bone; blue lines = maxillary sinus; pink lines = nasal cavity.

The rectangle lines were used to delineate and measure structures adjacent to the tooth using the “Lines” tool. Whenever the adjacent structure changed over a minimum distance of 1 mm, the current measurement was terminated, saved in “ROI Manager”, and a new measurement was generated for the subsequent structure (Figure 9). This process was repeated along the entire perimeter of the rectangle to quantify the length of the structures adjacent to the alveolus. The lengths of the lines representing adjacent structures were measured by a single observer using the “Measure” tool in the “ROI Manager”.

All measurements were recorded in an Excel spreadsheet. The individual lines representing the lengths of the adjacent structures were summarized as follows. For the intraalveolar crown, the length of each adjacent structure to the alveolus was calculated for each anatomical direction (buccal, oral, mesial, distal) by summing the lengths of all line segments of the respective structure in the corresponding direction. Additionally, the total length of each structure along the entire alveolar circumference was determined by adding the length of all corresponding line segments. Due to the angulation of the roots within the jaw, the section plane was not perpendicular to the tooth axis. Consequently, the edges of the roots in a section could not be unambiguously assigned to distinct directional positions (buccal, oral, mesial, distal). Therefore, the total lengths of each adjacent structure along the entire alveolar bone proper were recorded at the roots. The total lengths of each adjacent structure along the alveolar circumference could not be clearly assigned to either the distal or palatal root of the fused buccodistopalatal root. In these cases, the calculated lengths of each adjacent structure along the alveolar circumference were fully assigned to both the distobuccal and palatal roots.

The structures spongy bone, maxillary sinus, and nasal cavity were grouped as more vascularized bony structures.

The percentage ratio between the tooth dimension and the lengths of adjacent structures was calculated. For each anatomical direction (e.g., buccal), the total length of one structure was divided by the total length of the corresponding tooth direction. The percentage ratio between the cumulative length of each adjacent structure along the entire alveolar circumference and the entire alveolar circumference was determined.

### 2.3. Statistical Analysis

Descriptive statistical methods were used for data analysis. Mean values were calculated, and box plots were generated to illustrate measures of central tendency and dispersion, providing initial insights into the difference between the maxilla and mandible, between tooth positions, and age-related changes. The data were assessed for Gaussian distribution using the Shapiro–Wilk test (program Origin, Version 2025b, OriginLab Corporation, Northampton, MA, USA). If the data were normally distributed, a *t*-test was performed; otherwise, the Mann–Whitney U test was used. Significance was set at *p* < 0.05.

## 3. Results

A total of 188 maxillary and 204 mandibular sections from 72 cheek teeth were analyzed.

### 3.1. Comparison of the Peridental Compact Bone Distribution Between Maxillary and Mandibular Cheek Teeth Without Differentiating Between the Age Groups

**Figure 10:**
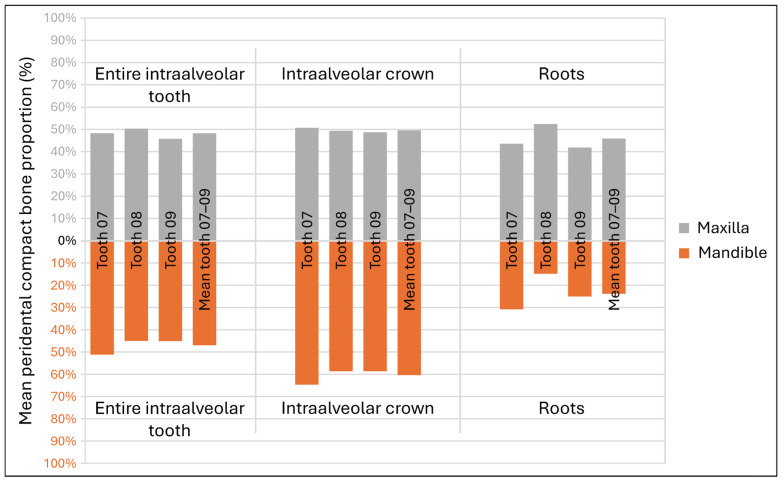
Comparison of peridental compact bone distribution between tooth positions (07, 08, 09) and mean of teeth 07 to 09 in the maxilla and mandible. Illustrated are the mean proportions (%) of peridental compact bone for tooth positions 07 to 09 and the mean of teeth 07 to 09 for different tooth regions (entire intraalveolar tooth, intraalveolar crown, roots) in the maxilla (gray) and mandible (orange). The values for entire intraalveolar tooth describe the mean of all intraalveolar crowns and roots. The values for roots represent the mean of all roots in the respective jaw.

The compact bone surrounding the intraalveolar crown was significantly increased (*p* < 0.05) in the mandible (60.3%) compared to the maxilla (49.5%) (Table 1, Figure 10). In contrast, the roots contained a significantly higher proportion of compact bone in the maxilla than in the mandible (*p* < 0.05). Within the maxilla, the proportion of compact bone between the intraalveolar crown (49.5%) and roots (45.8%) was nearly identical, corresponding to an approximate ratio of 50:50 between compact bone and more vascularized bony structures. The mandibular roots were surrounded by considerably less compact bone (23.7%) in comparison to the intraalveolar crown (60.3%). Comparable proportions of compact bone were observed around the mesial and distal roots in the mandible, as well as around the distal and palatal roots in the maxilla. In the maxilla, a higher proportion of compact bone was observed around the mesial root (55.6%) than around the distal (39.0%) and palatal (35.4%) roots. The entire intraalveolar tooth was surrounded by approximately 50% compact bone. However, the distribution of these structures differed between maxillary and mandibular intraalveolar crowns and roots.

### 3.2. Tooth Position-Related (07 to 09) Differences in Peridental Compact Bone Distribution in the Maxilla and Mandible

The tooth positions 07 to 09 in the maxilla and the mandible were examined in terms of their peridental compact bone proportion for the respective tooth section (Figure 10). The proportion of compact bone surrounding the entire intraalveolar tooth and the intraalveolar crown exhibited no significant differences between tooth positions 07 to 09 in the maxilla and mandible.

Small position-related differences were observed in the distribution of compact bone between the individual roots. The mean proportion of compact bone around the entire roots of tooth positions 07 and 09 did not differ significantly between the maxilla and mandible. In contrast, the entire root of tooth position 08 was surrounded by approximately 10% more compact bone in the maxilla and about 10–15% less compact bone in the mandible compared to tooth positions 07 and 09. The compact bone distribution showed nonsignificant variations among the individual roots at each tooth position in the maxilla and mandible.

A comparable pattern was observed when comparing premolars (07, 08) with molars (09).

### 3.3. Age-Related Changes in Peridental Compact Bone Distribution at the Intraalveolar Crown in the Maxilla and Mandible

**Figure 11:**
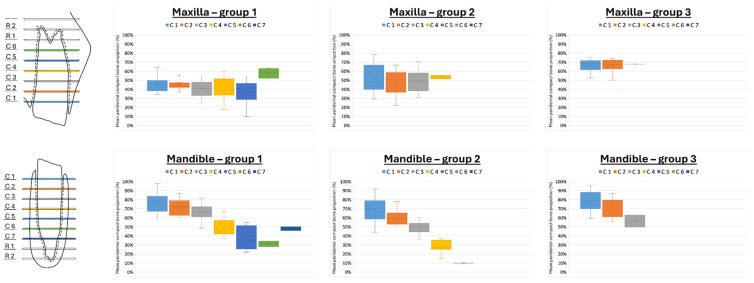
Age-related changes in the peridental compact bone distribution along the intraalveolar crown in equine maxillary and mandibular cheek teeth. The box plots show the percentage of peridental compact bone along the intraalveolar crown from the occlusal (C1) to the apical. The upper row represents the maxilla, the lower row the mandible. In each row are three diagrams, which correspond to the different age groups: left: Group 1, middle: Group 2, right: Group 3. Each box plot color represents a defined horizontal section: Light blue = C1, orange = C2, gray = C3, yellow = C4, medium blue = C5, green = C6, dark blue = C7.

In the maxilla (Figure 11), Group 1 and Group 2 horses exhibited a largely homogenous proportion of compact bone in the intraalveolar crown from the occlusal toward the apical direction. However, Group 2 horses showed greater variability. The compact bone proportion in Group 1 slightly decreased toward the apical section, followed by a small increase in the most apical section. Group 3 horses had a consistently higher and more uniform proportion of compact bone from the occlusal to the apical along the intraalveolar crown compared to Group 1 and Group 2.

In the mandible (Figure 11), the peridental compact bone proportion decreased from the occlusal to the apical in all three groups. A minor increase in the compact bone proportion was detected in the most apical section of Group 1. Horses in Group 3 displayed a higher proportion of compact bone across all horizontal sections compared to Group 1 and Group 2. The intraalveolar crown of old horses remained comparatively compact.

A more pronounced occluso-apical decrease in compact bone proportion was observed in the mandible compared to the maxilla. The changes in the maxilla were moderate, and values remained at a similar level. Group 3 consistently exhibited the highest proportions of compact bone in the maxilla and mandible.

## 4. Discussion

This study provides the first insights into the anatomical structure of the peridental region surrounding the equine cheek teeth. The results of the present study provide partial support for our hypothesis that anatomical differences are present in the peridental distribution of compact bone and more vascularized bony structures. The intraalveolar crown is surrounded by more compact bone in the mandible compared to the maxilla. There were no significant age-related variations between Group 1 and Group 2. Significant age-related differences were observed between Group 3 and Group 1 or Group 2.

The present study revealed that the total volume of peridental alveolar compact bone did not differ significantly between the maxilla and mandible. However, the intraalveolar crowns in the mandible were surrounded by more compact bone than in the maxilla, while the roots exhibited the opposite pattern. The human literature describes the mandible as being more compact, while the maxilla is comparatively spongier [1,25,26,27,28]. The results of the present study suggest similar jaw-dependent differences along the intraalveolar crown in horses. A direct comparison with human studies [1,25,26,27,28] is limited due to methodological differences (e.g., determination of the bone density or the thickness of cortical or cancellous bone) and anatomical distinctions (e.g., reserve crown in horses). Human literature often focuses on the alveolar bone, which is defined as the entire tooth-bearing or formerly tooth-bearing part of the jaw [26,27,28], whereas this study examined the ABP and its adjacent peridental structures.

To date, two clinical studies have examined which tooth positions are most commonly affected by post-extraction complications [18,19]. The results of these studies demonstrate slight variations: Gergeleit et al. [18] reported that the mandibular cheek teeth position 07 and 09 are most frequently affected, whereas Kennedy et al. [19] identified mandibular cheek teeth positions 06 to 08 as being most frequently observed. The results of the present study showed that the amount of peridental compact bone did not differ between the different tooth positions 07 to 09. Small differences were observed between tooth positions at the roots. These differences may reflect the morphological heterogeneity in the shape and orientation of the roots. Teeth 06, 10, and 11 should be included in a further study with larger sample size in order to determine whether the peridental bone structure differs in positions that are less prone to complications following tooth extraction.

The peridental compact bone along the crown of the tooth decreased in thickness in the mandible from the occlusal to the apical of the root in all age groups compared to the maxilla. An increase in peridental compact tissue was observed solely in Group 1 in the most apical section. Nonetheless, the reliability of this finding is low due to the small sample size.

Variations in the peridental bone structures around the tooth crown may result from anatomical and biomechanical factors. Jawbones continually undergo remodeling processes, which are initiated by a variety of factors, including masticatory forces acting on the teeth [29,30]. Masticatory forces are absorbed by the PDL and subsequently transferred to the jawbone [31]. Mechanical forces form invisible trajectories (lines of force or tension) in the jawbone [1,32]. The trabeculae of the spongy bone align along these trajectories to absorb and transmit the forces acting on the jaw, like pressure and tension [1,32]. Cordes et al. [31,33] observed that the compression and tensile stresses that occur during mastication in horses appear on different sides of the maxilla and mandible. In the maxilla, compressive stresses occur palatally in the region of the alveolar ridge and buccally near the root apex, whereas tensile stresses are observed buccally at the alveolar ridge and palatally near the root apex. The mandible exhibits a reversed pattern [31,33]. These variations in trabecular orientation and thickness could explain the differences in cancellous bone volume between the maxilla and mandible observed in our study. Repeated higher stresses lead to a gradual thickening of the trabeculae. Consequently, the progressive thickening of the trabeculae and the resulting narrowing of the spaces between them may lead to a partial coalescence of adjacent trabeculae, giving the bone a more compact appearance.

Tooth shape may also contribute to these differences. The maxillary cheek tooth exhibits a greater attachment surface for the PDL due to its squarer shape and greater volume [34]. This facilitates a more uniform distribution of masticatory forces and may result in a less compact bone structure. The mandibular cheek tooth has a smaller surface area and volume [34] and may require more compact bone (especially occlusally) to absorb masticatory forces. It could be hypothesized that the observed variation in compact bone content between the maxilla and mandible may be attributable to differences in tooth shape.

It has been reported by dental practitioners that the oral extraction of mandibular cheek teeth is more difficult and requires greater force than the extraction of maxillary cheek teeth. In the preliminary study by McGinley and Reardon [35], the forces applied during maxillary and mandibular dental extraction were quantified. A comparison between maxilla and mandible was absent; however, it is evident that numerous factors influence the required forces, including the area of periodontal attachment or the position of the fulcrum. In cases where the fulcrum is positioned in closer relation to the tooth being extracted, higher elevation forces are required. Dixon [16], Gergeleit et al. [18], and Kennedy et al. [19] previously hypothesized that the increased extraction forces may result in (micro)fissures and local ischemia due to blood vessel damage, which can promote the formation of sequesters. During extraction, the alveolar bone undergoes partial or complete fragmentation, resulting in compromised blood supply to the affected fragments. Fragments that are not manually removed remain in the dental socket. While fragments in the maxillary empty alveolus may more easily drop into the oral cavity, fragments in the mandible remain within the alveolus due to gravitational forces. In the case that these fragments remain in the dental socket, the probability of subsequent sequestration is increased [36]. The more rigid bone structure observed in the occlusal area in this study may contribute to the enhanced difficulty in loosening during the process of extracting cheek teeth. An unfavorable extraction angle could exacerbate this further, requiring higher forces and causing greater damage to the peridental region.

Sequestration is considered to result from a combination of local ischemia and infectious agents [20,21,22,37]. Local factors, including blood supply and formation of the blood clot, may affect wound healing following tooth extraction [15,20,38,39,40]. Reduced bleeding into the alveolus may impair wound healing by limiting revascularization, tissue regeneration, and elimination of infectious agents. Furthermore, the accumulation of infectious exudate has the potential to hamper the local immune response, revascularization of bone fragments, and wound healing [20,41]. Bone fragments that cannot be resorbed or revascularized, are encapsulated as sequesters [20,22,41].

The presence of a small, infected, or prematurely resorbed blood clot within the alveolus after tooth extraction can result in delayed wound healing [42,43,44] and promote the sequestration of bone fragments [20,38,39,40]. A portion of the alveolar bone and/or ABP may remain exposed, where a so-called dry socket can develop [43,44]. The bone undergoes necrosis and may migrate into the wound cavity as a fragment, resulting in the formation of a sequestrum. The results of the present study indicate that more vascularized bony structures are distributed evenly along the crown and roots in the maxilla, comprising approximately 50% of the peridental structures. This distribution offers numerous potential sources of bleeding along the entire intraalveolar tooth, which may facilitate sufficient bleeding into the dental socket. In contrast, the mandible shows a progressive increase in more vascularized bony structures from approximately the mid-portion of the intraalveolar crown toward the apical region. The results of the study suggest that the tissue distribution in the mandible may be associated with inadequate bleeding into the extraction socket. In combination with other factors, such as the anatomical position of the mandibular alveoli, which restricts physiological drainage into the oral cavity, infectious agents, or the efficiency of immunology response, this may promote the sequestration of bone fragments [20,38,39,40].

To summarise, it can be stated that post-extraction complications should be considered multifactorial. In addition to the anatomy of the peridental tissues, factors such as the duration of tooth extraction, gravitational forces, insufficient bleeding into the empty alveolus, the presence of infectious agents, the immunological response, the shape of the tooth, and the number and size of ABP fragments can impair wound healing and promote the formation of sequestra.

Gergeleit et al. [18] and Kennedy et al. [19] reported that post-extraction complications occur most frequently following mandibular cheek teeth extraction in younger horses, and teeth 07 and 09 [18] or teeth 06 to 08 [19] are most commonly affected. Sequestration has been identified as the most frequently reported complication.

The present study demonstrated that horses in Group 3 tended to exhibit an increased amount of peridental compact bone along the intraalveolar crowns of the maxilla and mandible compared to Group 1 and Group 2. Older horses showed a reduction in tooth surface, denser and tighter PDL fibers, and a more perpendicular angle of inclination of the PDL fibers toward the tooth surface [45,46]. This modification process can be interpreted as a compensatory mechanism. We hypothesized that increased peridental compact bone in older horses provides the thicker PDL fibers with a more resilient and greater anchorage surface. Nevertheless, the risk of periodontal disease increases with age, which can impair the anchoring of teeth, allowing teeth in older horses to be extracted more easily with lower elevation force [35]. In contrast to the results of Gergeleit et al. [18], the present study did not reveal any abnormalities in the peridental region of the teeth that could explain the frequent occurrence of sequestration in younger horses.

The present study was conducted with the aim of characterizing and comparing the alveolar structure in the immediate surrounding area of the ABP. The methodology developed for the geometric simplification of the alveolar bone shape in conjunction with the defined classification of spongy bone enabled an efficient evaluation. Nevertheless, it must be noted that this methodology merely provides an approximation to the complex biological reality. The peridental bone was assessed at a distance of 1 mm from the ABP, which enabled a targeted investigation of the peridental bone structure that was damaged during tooth extraction. The definitions of spongy and compact bone were developed based on macroscopic criteria and have proven practicable and consistently applicable. However, it should be noted that small individual pores may correspond to blood vessels or nerve tracts, potentially leading to an overestimation of the spongy bone proportion. Furthermore, local structural variations, such as the presence of thick trabeculae or an accumulation of small individual pores in the compact bone, have the capacity to influence the classification of the tissue into the defined tissue types.

This study should be considered a preliminary study due to the limited sample size. Further studies with larger sample sizes are necessary to verify the results, especially regarding tooth positions 06, 10, and 11, to obtain a comprehensive picture of regional differences in the peridental region. Combining these studies with histological validation and micro-CT (µCT) would improve tissue classification accuracy.

The present results provide important basic information on the alveolar structure, which should be further investigated using µCT images and finite-element analyses. In particular, finite element analysis has the capacity to simulate the forces acting during extraction, thereby facilitating a more profound comprehension of the functional significance of alveolar microstructure. Moreover, it can be utilized to derive conclusions about postoperative complications following tooth extraction.

## 5. Conclusions

The results of this descriptive anatomical study provide a novel anatomical characterization of the peridental region of equine cheek teeth. The findings offer partial support for the hypothesis that structural differences in the peridental region may influence the occurrence of extraction-related complications. Maxillary teeth were found to be surrounded by a more balanced distribution of compact bone and more vascularized bony structures. In contrast, a higher proportion of compact bone was observed in the occlusal region of mandibular cheek teeth, with a decrease in the apical region. These anatomical variations may influence the mechanics of extraction and the subsequent development of complications. The compact bone is more prone to microfissures and fractures than spongy bone, which increases the risk of sequestration. However, the previously reported higher incidence of postoperative complications following tooth extraction, particularly sequestration in younger horses, cannot be attributed solely by the structure of peridental tissues.

## Figures and Tables

**Table 1:** Comparison of the peridental compact bone distribution between maxilla and mandible (entire dataset, *n* = 392 horizontal sections).

Anatomical Section		Peridental Compact Bone Mean	
		Maxilla	Mandible
Entire intraalveolar tooth		48.1%	46.9%
Intraalveolar crown		49.5%	60.3%
Roots		45.8%	23.7%
Roots	mR	55.6%	25.8%
	dR	39.0%	21.4%
	pR	35.4%	

The values represent the overall results for all examined teeth, regardless of tooth position or age group. The entire intraalveolar tooth describes the mean of all intraalveolar crowns and roots. The results from roots are reported as a combined mean for all roots. Abbreviations: mR = mesial or mesiobuccal root, dR = distal or distobuccal root, pR = palatal root.

## Data Availability

The raw data supporting the conclusions of this article will be made available by the authors on request.

## Abbreviations

The following abbreviations are used in this manuscript:

µCT	micro-computed tomography
ABP	alveolar bone proper
b	buccal
C1–C7	numbered anatomical section of intraalveolar crown
CT	computed tomography
d	distal
dR	distal (mandibular) or distobuccal (maxillary) root
m	mesial
mR	mesial (mandibular) or mesiobuccal (maxillary) root
o	oral
PDL	periodontal ligament
pR	palatal (maxillary) root
R1, R2	numbered anatomical section of roots
